# Cement Augmentation of the Blade in Proximal Femoral Nailing for Trochanteric Fractures in Elderly Patients: A Retrospective Comparison of Mechanical Stability and Complications

**DOI:** 10.3390/jcm14217469

**Published:** 2025-10-22

**Authors:** Zoltan Cibula, Marian Grendar, Diaa Sammoudi, Milan Cipkala, Marian Melisik, Maros Hrubina

**Affiliations:** 1 Jessenius Faculty of Medicine in Martin, Comenius University in Bratislava, 036 59 Martin, Slovakia; cibulazolo@gmail.com (Z.C.); marian.grendar@uniba.sk (M.G.); melisik2@gmail.com (M.M.); 2 Department of Orthopaedic Surgery, University Hospital Martin, Kollarova 2, 036 59 Martin, Slovakia; dia.samoudi@gmail.com (D.S.); milan.cipkala@gmail.com (M.C.)

**Keywords:** trochanteric fractures, proximal femoral nail, spiral blade, cement augmentation

## Abstract

**Background**: Cephalomedullary nails are the standard treatment of trochanteric fractures, and some implants with a perforated blade allow augmentation with bone cement to increase mechanical stability. The study compares the results of PFNA and TFNA implants (DePuy Synthes) with or without cement augmentation of the blade. **Methods**: A retrospective study evaluated 219 trochanteric fractures. The study included 59 men (27%) and 160 women (73%), with a mean patient age of 82 years. The most common fractures were type 31A2 (56%), followed by type 31A1 (25%) and type 31A3 (19%). The monitored parameters were evaluated from anteroposterior and axial images of the proximal femur and pelvis. TAD, blade position, lateral blade prominence, fracture varus, and cut-out were evaluated. **Results**: Cement-augmented blade implants (CABs) in 68 patients (31%) and cement-free implants (NCABs) in 151 patients (69%) were used. The average age difference between the groups was 7 years (CAB 86.07 ± 5.85 and NCAB 79.13 ± 8.48). CABs were used more frequently in women (60 cases) than in men (8 cases). Blade position was optimal in 68% of cases and suboptimal in 32%. The risk of varus deformities was not statistically significantly affected by the blade position. The statistical significance of CABs for reducing the risk of varus deformities in stable fractures (*p* = 0.396) or unstable fractures (*p* = 0.101) was not confirmed. The average varus angulation during treatment was 2.57° (CAB 2.53° and NCAB 2.67°). A varus deformity greater than 10° was confirmed in 8 eight patients (3.7%) and cut-out in three patients (1.4%). All patients with cut-out were in the NCAB group. Cement leakage occurred in two cases and was asymptomatic. One case of deep infection, lateral blade prominence, and avascular necrosis (AVN) were recorded. **Conclusions**: Cement augmentation of the blade did not significantly reduce varus deformity in this cohort, regardless of blade position of fracture stability. CABs may prevent cut-out in specific subgroups, but this requires further investigation.

## 1. Introduction

The increasing age of patients is associated with a rising incidence of proximal femur fractures [1]. These occur predominantly in patients over 65 years of age, and the severity of these injuries is highlighted by the fact that they are one of the leading causes of death in the elderly population worldwide [2,3].

The vast majority of these fractures are associated with reduced bone quality and occur in a group of frail patients prone to repeated falls. Proximal femur fractures include intracapsular fractures of the femoral head and neck, which are mainly an indication for arthroplasty in older patients. Extracapsular fractures include trochanteric and subtrochanteric fractures of the proximal femur. Trochanteric fractures are very common in old age and account for more than half of all proximal femur fractures [4]. In the past, these types of extracapsular fractures of the proximal femur were surgically stabilised using various methods such as wires, screws, or plates.

Currently, the method of choice is the use of cephalomedullary nails. For stable fractures, DHS plates can also be used, sometimes in combination with trochanteric stabilising plates (TSPs). Despite advances in the design of cephalomedullary nails, there is still a risk of failure of surgical treatment of proximal femur fractures, especially in older patients. Over the last decade, two cephalomedullary implants have been used at the Orthopaedic Clinic at the Martin University Hospital for the treatment of trochanteric fractures. These are the PFNA and TFNA proximal femoral nails. Both implants are indicated for the surgical treatment of simple and complex trochanteric fractures in elderly patients with poor bone quality. The implants allow for simultaneous cement augmentation of the blade in the femoral head area to increase the stability of osteosynthesis.

Biomechanical experiments have shown higher stability of the osteosynthesis with the use of cement augmentation of the femoral neck blade. More test cycles until mechanical failure of the osteosynthesis, better rotational stability, and higher pullout strength were achieved on the cadaveric models [5].

The first promising results of cement-augmented blades were presented by Kammerlander et al. [6]. In recent years, few studies argued that cement augmentation could reduce the incidence of cut-out phenomenon, especially in osteoporotic bones [7]. The other authors presented similar results of both groups (with and without augmentation) focused on complications and revisions [8].

Therefore, we have decided to present our results of cement augmentation of the proximal femoral nail in the treatment of the osteoporotic proximal femoral fractures.

The aim of this study was to compare the outcomes of patients with and without cement augmentation of the cephalomedullary nail.

We hypothesised that cement augmentation of the blade would reduce varus deformity, cut-out, and reoperation rates in osteoporotic pertrochanteric fractures treated with PFNA/TFNA.

## 2. Materials and Methods

This is a retrospective study following patients older than 63 years with extracapsular fractures of the proximal femur type 31A (AO-OTA classification), treated from September 2017 to December 2024. The cohort included patients after falls with low-energy trauma mechanisms, and patients were treated surgically using intramedullary osteosynthesis. The cohort did not include patients with pathological fractures, patients with proximal femur fractures other than pertrochanteric (31A1,2) or intertrochanteric (31A3), patients younger than 63 years of age, or patients with high-energy trauma. Patients who had undergone previous surgery in the area of the injured hip joint or who had bone dysplasia were excluded. Patients in whom we were unable to adequately assess the evaluated parameters by radiological examination and patients in whom X-ray evaluation was not possible until the fracture had healed for various reasons, including death, were not evaluated. One of two implants with a perforated blade was used for the osteosynthesis of trochanteric fractures, namely the PFNA implant (Proximal Femoral Nail Antirotation, DePuy Synthes) or the TFNA implant (TFN-ADVANCE Proximal Femoral Nailing System, DePuy Synthes). The operations were performed according to standard surgical procedures for each of the femoral nails. When using cement augmentation of the blade, we used TRAUMACEM V+ bone cement (Augmentation System, DePuy Synthes). The use of cement augmentation of the blade was chosen individually, taking into account the age, type of fracture, and above all, bone quality. These injuries were treated by several physicians who took into account the individual use of cement for fracture instability based on the course of the fracture lines, its comminution, size, and direction of fragment displacement. The bone quality, mainly during surgery, when inserting guide wires, pins, and especially blades was assessed.

Radiological parameters from standard projections—anteroposterior images of the pelvis, images of the proximal femur in anteroposterior projection (anterior/posterior—AP), and axial projection (axial Lauenstein) were analysed. During the initial examination of the patient after the injury, a clear image of the pelvis and proximal femur was performed. After surgery and then 6 months after surgery, the X-ray measurements from the AP and axial projections of the proximal femur were evaluated. The 6-month interval was sufficient for the fracture to heal in all patients. In patients with significant complications requiring reoperation, 6-month measurements were not evaluated.

**Figure 1:**
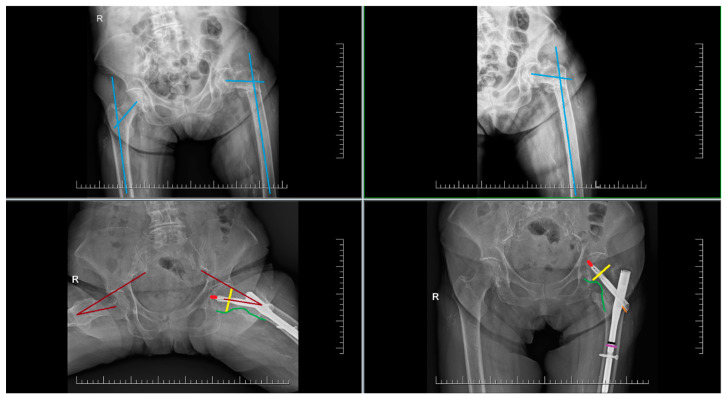
Standard projections and evaluated parameters: CCD angle (blue), TAD (red), blade course/position (yellow), lateral blade prominence (orange), anteversion (burgundy), and anterior cortical line and medial cortical line (green).

From clear X-ray images of the pelvis, the collum-diaphyseal (CCD) angle on the contralateral side before surgery was evaluated (Figure 1). From the same X-ray images, the type of fracture according to the AO/OTA classification was determined. After surgery, the following in AP projections were evaluated: CCD angle, fracture shortening, the distance between the tip of the blade and the subchondral bone, known as TAD (tip-apex distance) in two projections, the distance between the lateral end of the blade and the nail, and the position/course of the blade in the femoral head. TAD was calculated according to Baumgaertner’s description [9]. The position of the blade in the femoral head was determined based on the work of Cleveland [10]. The quality of reduction by evaluating the medial cortical line was assed, where we assessed both the disrupted continuity and displacement of fragments or the presence of a gap along this line. If the dislocation or gap was greater than 3 mm, the reduction in the area of the medial cortical line (Adam’s arc) was assessed as non-anatomical.

Radiographic measurements (CCD, TAD, blade position, lateral blade prominence, and varus/valgus angles) were performed by an experienced radiologist/in orthopaedics and trauma surgery/, which was not included in the study. He performed all measurements. A subset of 20 randomly selected radiographs was re-evaluated after two weeks (by D.S. and M.G.) to assess intraobserver and interobserver reliability using intraclass correlation coefficients (ICCs). ICCs ˃ 0.80 were considered excellent.

After surgery, the following lateral projections were evaluated: anteversion of the femoral neck and the position of the blade in the neck and head of the femur. The quality of reduction by repositioning the anterior cortical line and was assessed, in this case, the disrupted continuity and gap between the fragments was evaluated. If the dislocation was more than 3 mm, the position was assessed as non-anatomical.

Patients were operated on in the supine position using traction on an extension table. The surgical technique for implanting the nail was performed according to the manufacturer’s surgical manual. After inserting the guide wire into the neck and checking its position in the AP and lateral projections, the lateral cortex was opened by drilling. The neck or head of the femur was not drilled and implanted the blade. When using cement augmentation, a contrast agent was first applied and then injected the cement using the standard technique. During TFNA implantation, the locking mechanism in the wedge was tightened and then turned it back half a turn (180°) to allow compression of the fracture. The aim was to start rehabilitation with the help of a physiotherapist and walking on the first postoperative day. Patients used German crutches or a G-apparatus during rehabilitation, depending on their tolerance.

The study does not assess functional outcomes (mobility, pain).

The data were reviewed and analysed using the Jamovi software (version 2.6.44.0), which is based on R [11,12]. Group comparisons were performed using Student’s *t*-test for the comparison of means. Categorical data were compared with chi-square test and Fischer’s exact test. Statistical differences were considered to be significant when the *p* value was <0.05.

The data analyses (by performing multivariate regression modelling) were realised with the following: logistic regression for binary outcomes (e.g., change in angle [yes/no]) and linear regression for continuous outcomes (e.g., post-operational TAD).

## 3. Results

A total of 219 trochanteric fractures were retrospectively evaluated. Of these, eight patients underwent surgery for trochanteric fractures in both lower limbs during this period. The average age of the patients was 82 years (63–98 years). The patient group included 59 men (27%) and 160 women (73%). Based on the AO-OTA classification, patients with 31A1, 31A2, and 31A3 fracture types were monitored.

The most common fractures in the patient group were 31A2 fractures (56%), followed by 31A1 fractures (25%), and, less frequently, 31A3 fractures (19%). Stable fractures (31A1 and 31A2.1) accounted for 35% of patients in the study group and 65% of patients had unstable fractures (31A2.2, 31A2.3, and 31A3).

Implants with cement augmentation of the blade (CAB) in 68 patients (31%) and implants without cement (NCAB) in 151 patients (69%) were used. The average age difference between patients with cement and non-cement augmentation was 7 years (CAB 86.07 ± 5.85 and NCAB 79.13 ± 8.48).

Of the total number of patients, a combination of PFNA and cement in 45 women (21%) was used compared to 7 men (3%) when using cement augmentation. In cases of osteosynthesis using TFNA, cement augmentation in 15 women (7%) compared to 1 man (0%) was used. Cement augmentation of the blade was therefore more frequently used in women, where we used it in 60 cases in women and 8 cases in men (Table 1).

**Figure 2:**
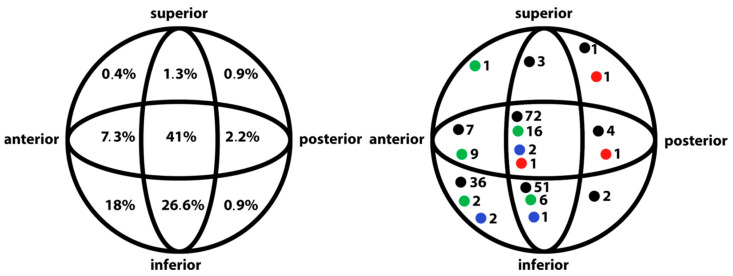
Position of the blade in the femoral head. (**Left**): percentage distribution of blade positions in the patient group. (**Right**): the position of the blade in cases without varotization (black), with varotization of 5–10° (green), with varotization of ˃10° (blue) and cut-out (red).

The course of the blade in the femoral head was evaluated. The central–central and central–inferior positions were considered to be optimal blade positions. Based on this, we rated 68% of the positions as optimal. The most common non-optimal positions were inferior–anterior (18%) and central–anterior (7.3%) (Figure 2).

In the monitored group, the average TAD value after surgery was 19.1 mm and after healing was 18.85 mm. With cement augmentation, the average TAD value after surgery was 17.78 mm and after healing was 17.49 mm. Without cement augmentation, the TAD was 19.7 mm after surgery and 19.45 mm after healing. The average difference in distances measured after surgery and at the time of fracture healing was 0.32 mm (*p* = 0.003). The statistical significance of the difference between the two distances based on the use or non-use of cement augmentation of the blade (*p* = 0.751) or when comparing fracture stability/instability (*p* = 0.399) was not confirmed.

**Figure 3:**
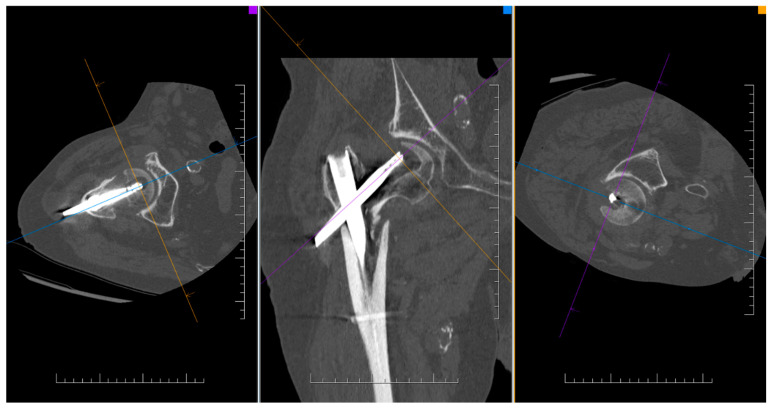
CT finding of osteosynthesis failure using TFNA without cement augmentation of the blade and with cut-out.

One of the complications of treatment is the lateral prominence of the blade (Figure 3). Statistical analysis showed that the average difference between the BN-BE (blade nail–blade end) distance immediately after surgery and after 6 months was 5.52 mm (*p* = ˂0.001).

Varotization of the fracture is expressed in the evaluated group of patients by the difference in CCD angles during treatment. The average varotization of fractures was 2.57°. The average varotization of fractures in the case of cement augmentation was 2.53, and in the group without cement augmentation, 2.67. No statistical difference in the risk of varotization was confirmed in patients with or without cement augmentation of the blade (*p* = 0.789) (Appendix A).

**Figure 4:**
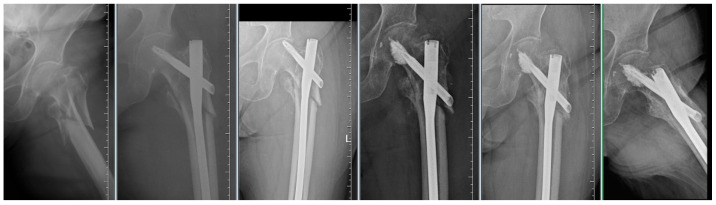
Failure of osteosynthesis of a comminuted trochanteric fracture treated with TFNA. Reosteosynthesis was performed with cement augmentation of a new blade in the central–inferior position; image shows healed fracture.

**Figure 5:**
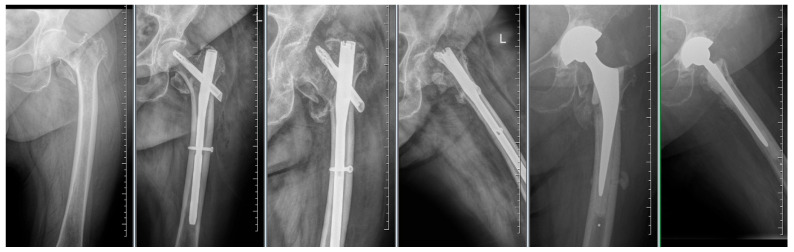
Failure of osteosynthesis and cut-out of the blade after osteosynthesis using TFNA. Extraction of TFNA and implantation of a total hip endoprosthesis.

By evaluating the X-ray parameters, eight patients in the cohort in whom postoperative varus deformities of the femoral neck were greater than 10° were founded. Four patients did not have cement augmentation and four had cement augmentation of the nail. In three cases, the blade cut-out and, thus, failure of osteosynthesis was identified. In two women and one man with blade cut-out, a TFN-A implant without cement augmentation was implanted (Figure 3, Figure 4 and Figure 5). In these three cases, the medial cortical line and anterior cortical line were repositioned within 3 mm of dislocation at the anatomical interface. The blade cut-out in the group with cement augmentation was not observed.

A postoperative varus deformity of 5–10° was recorded in a total of 35 patients. Without cement augmentation, there were 31 patients in this group and with cement augmentation there were four patients. Fifteen patients were operated on using PFNA and 20 patients using TFNA. Of these, stable fractures according to the AO/OTA classification included 12 cases in this set, and 23 patients had unstable fractures. The risk of varus deformity was not statistically significantly affected by optimal or suboptimal blade position (*p* = 0.690).

Statistical evaluation to assess the effect of cementing on reducing the risk of varotization in patients based on blade position and fracture stability was used. Despite the use of cement augmentation in cases with optimal blade position, patients did not show a statistically significant reduction in the risk of varotization (*p* = 0.179). This was also not confirmed in patients with suboptimal blade position and the use of cement augmentation (*p* = 0.245). The reduction in the risk of varus deformity with cement augmentation was not statistically significant in either the group of patients with stable fractures (*p* = 0.396) or in the group of patients with unstable fractures (*p* = 0.101).

The CI was 95% and clinical relevance is significant.

Age and gender were not significant predictors in any of the regression models.

Complications included one case of deep infection requiring complete removal of the osteosynthetic material and another case of lateral blade prominence, which was an indication for its removal. In one case, avascular necrosis of the femoral head was identified.

Cement leakage was observed in two cases. In the first case, the leak was in the area of the femoral neck, and in the second case, in the area of the femoral head after drilling through the head with a guide wire. In two cases, a fracture of the lateral cortex was identified, which occurred during the insertion of the nail.

## 4. Discussion

The stability of osteosynthesis of trochanteric fractures depends on several factors. These include bone quality, fracture morphology (stability/instability), quality of reduction, correct choice of implant, and, in the case of cephalomedullary nails, the position of the screw or blade in the femoral head. The quality of the reduction and the position of the implant can be influenced by the surgical technique. Our clinical experience and study results confirm the key importance of fracture reduction and the restoration of anatomical relationships [13,14,15]. For reduction, it is important to check the continuity of the cortical lines, medial and anterior cortical support, and the CCD angle. Kristan et al. evaluated reduction in lateral projection as the most important prognostic factor for a favourable radiological outcome. Several biomechanical and clinical studies confirm the importance of anterolateral cortical support. The posterior cortex is significantly more frequently comminuted (60.4%) compared to the anterior cortex (3%), and it is precisely the comminution of the anterior cortex that may be a predictor of failure [16]. Chang et al. proposed the concept of anteromedial cortical support, which provides secondary stability and promotes bone healing of the fracture by supporting the anteromedial cortex of the femoral neck fragment and the distal fragment [17]. It is important to recognise certain irreducible trochanteric fractures and, if necessary, use open reduction and fixation.

It is necessary to avoid varus deformity or translation, but excessive valgus and medial opening of the fracture line may also be problematic for healing. In our series, there were 20 patients (9%) with an initial reduction in varus deformity greater than 5°. In varus deformity greater than 5°, 43 fractures (19%) healed. In 28 patients (13%), initial reduction was in valgus deformity and was greater than 5°. In a group of 313 patients with intertrochanteric fractures treated with cephalomedullary nail osteosynthesis (PFNA Synthes), a varus deformity greater than 5° was found in 6.5% of cases [18]. Several surgical techniques can prevent this deformity. Thorough reduction and using the correct entry point are important, but drilling and nail insertion can also accentuate varus deformity. The correct trajectory is essential for maintaining reduction during cephalomedullary nailing of trochanteric fractures [19]. The design and curvature of current implants also help to avoid varus deformity. Fractures with a significant varus position are also risky for correct implant placement and require restoration of the anatomical position. The position of the blade itself is therefore significantly influenced by fracture reduction and surgical technique. Simmermacher evaluated the position of the PFNA implant blade in the femoral head and found that in a group of 70 patients, the position was good in 32.9%, acceptable in 47.1%, and not optimal in 20%. Four patients treated with PFNA were recommended for reoperation due to complications associated with the implant and fixation. Osteosynthesis using PFNA did not lead to a significant improvement in blade position in the femoral head compared to PFN, but it did reduce the risk of complications and the need for reoperation [18]. The position of the blade can be assessed on an anterior–posterior and axial X-ray image by its course in nine quadrants. In the AP projection, it is classified as superior, central, or inferior, and in the axial projection, as anterior, central, or posterior. The blade implantation can be considered optimal in both projections in the central–central or central–inferior position. For comparison, Bohringer evaluated the position of the TFNA blade as optimal in 82.4% of 620 patients [20]. In our cohort, we evaluated the position as optimal in 68% of cases. The most common non-optimal position was inferior–anterior in 18% of cases and central–anterior in 7.3% of cases.

The position of the blade in the femoral head can be expressed by the course of the blade in two basic planes and also as TAD, i.e., tip–apex distance. This distance was described by Baumgartner et al. as the sum of the distance from the tip of the blade/screw to the top of the femoral head in the anterior–posterior and lateral projections [9]. To ensure sufficient strength, it is important that the end of the blade ends in the trabecular bone, and it is optimal to insert the blade in the axis of the neck and head of the femur or slightly below it. The distance between the end of the blade and the level of the subchondral bone is approximately 10 mm.

Complications of trochanteric fracture treatment using cephalomedullary implants include malposition, varus collapse, cut-out, pull-out, and cut-through. These complications are not rare and occur in 4–7% of cases [21]. The occurrence of mechanical failure in patients is associated with a high rate of revision surgery, functional impairment, and mortality. Mortality after revision surgery is up to 56% within one year [22]. Mechanical failure of the blade (cut-out) through the femoral head occurs in various studies at an incidence of between 1.8 and 16.5%. The risk of cephalomedullary nail failure also varies depending on the type of implant used. Biomechanical studies report higher blade resistance in TFNA implants compared to screws [23]. However, there are also studies that report an increased incidence of cut-out when using blades compared to screws in TFNA implants. The incidence of fixation failure in connection with TFNA implants is reported to be between 2 and 7% of cases [24,25]. A higher degree of mechanical stability of osteosynthesis of some cephalomedullary nails can also be achieved with cement augmentation of the blade in the femoral head. Erhart et al. tested the anchoring of PFNA blades augmented with bone cement in eight freshly frozen femoral heads and demonstrated increased rotational stability and higher pull-out strength [26]. The use of cement augmentation is indicated in several studies in cases of poor bone quality, suboptimal fracture or blade positioning in the femoral head, or, for example, for the treatment of unstable fractures. These are precisely the cases where there is a higher risk of failure of osteosynthesis of trochanteric fractures. Cement augmentation does not increase the risk of postoperative complications or mortality. Its use can prevent failure and cut-out of the blade from the femoral head [27].

This study is based on a comparison of the results and incidence of complications in the treatment of trochanteric fractures with two proximal femoral nails (PFNA and TFNA) with and without the use of blade cementation. The surgical procedure was performed by several physicians, but we nevertheless attempted to determine the significance of blade cement augmentation. The results of our treatment with the results of other published studies were compared.

In a 2018 study, Kammerlander et al. followed a group of 223 patients (105 with augmentation and 118 without augmentation). They found that six patients from the group without cement augmentation had to undergo further surgery due to mechanical failure of osteosynthesis. In contrast, no failure occurred with cementing [6]. From our patient group, we can also conclude that mechanical failure cut-out phenomenon did not occur in the group of patients with cement augmentation. A patient with a metastatic fracture of the proximal femur was excluded from the sample, in whom we also observed osteosynthesis failure and who required subsequent conversion to total endoprosthesis.

Schneider et al. followed 264 patients in their retrospective study. The group included 18 patients (6.8%) who underwent further surgical procedures. No complications in the sense of cut-out occurred in the group of patients with PFN-A cement augmentation. Compared to the group without cement use (9%), there was a lower percentage of reoperation in patients with cement augmentation (2.3%) [28].

Konrad Schuetze, in his 2021 paper, presents a set of 152 patients who did not experience mechanical failure of osteosynthesis after 6 months of follow-up. The cement augmentation of the blade is safe and does not increase postoperative complications or mortality. This technique can prevent mechanical failure [27].

Cement augmentation should reduce varus deformity and reduce failure; however, in this cohort, augmentation did not significantly reduce varus risk overall.

Our study has its limitations. It is a retrospective study with a short follow-up of the incidence of mechanical complications in the treatment of trochanteric fractures. Being retrospective, causality cannot be established. Nevertheless, studies agree that six months is sufficient time for most of these fractures to heal, which was also confirmed by our follow-up. The follow-up period of at least 6 months after surgery was met by X-ray evaluation in the group of patients followed, with the exception of patients with complications requiring reoperation due to mechanical failure of the implant. The study primarily focused on evaluating differences in the incidence of complications with or without cement augmentation in osteosynthesis with PFNA and TFNA implants. Clinical parameters or patient satisfaction assessed by range of motion, VAS (visual analogue score) or, for example, HHS (Harris hip score) were not evaluated. The observed differences in some radiographic parameters should be interpreted with caution due to the retrospective design.

## 5. Conclusions

Cement augmentation of the blade did not significantly reduce varus deformity in this cohort, regardless of blade position of fracture stability. CABs may prevent cut-out in specific subgroups, but this requires further investigation.

The results show that cement augmentation of the blade in older patients, and more often in women was used. Although the optimal blade positioning was not achieved in one third of cases, the risk of varus deformity was not statistically significantly affected by this positioning. But augmentation may still prevent cut-out. The varus deformity of more than 10° in eight patients, of whom three were without cement augmentation, had cut-out was observed. This complication necessitated reoperation with material extraction and hip replacement. The cement leakage itself, which was observed in two cases, was not clinically significant.

## Figures and Tables

**Table 1:** Numbers of performed surgeries with or without cement augmentation.

**IMPLANT_USED CEMENT**	**GENDER**	**COUNTS**	**% OF TOTAL**
**PFN-A_NO**	M F	22 47	10 21
**PFN-A_YES**	M F	7 45	3 21
**TFN-A_NO**	M F	29 53	13 24
**TFN-A_YES**	M F	1 15	0 7

## Data Availability

The original contributions presented in this study are included in the article. Further inquiries can be directed to the corresponding author.

## Abbreviations

The following abbreviations are used in this manuscript:

PFNA	Proximal femoral nail antirotation
TFNA	Trochanteric femoral nail antirotation
TAD	Tip-apex distance
AP	Anteroposterior
CAB	Cement-augmented blade implant
NCAB	Cement-free implant
AVN	Avascular necrosis
DHS	Dynamic hip screw
TSP	Trochanteric stabilising plates
CCD	Collum-diaphysis angle

## Appendix A

**Table A1:** X-ray specification and statistical evaluation of both groups of patients.

Variables		CAB	NCAB	*p*-Values (s/ns)
Patients		68 (31%)	151 (69%)	
Age (years)		86.07	79.13 ±8.48	
Sex		f: 60 (27.4%) vs. m: 8 (3.6%)	f: 100 (45.6%) vs. m: 51 (23.3%)	
AO classification				
31A1		20 (9.1%)	34 (15.5%)	
31A2		31 (14.1%)	92 (42%)	
31A3		17 (7.76%)	25 (11.4%)	
				
Tip-apex distance PO (mm)-mean		17.78	19.7	
Tip-apex fistance PO (mm)-median		17.19	19.39	
Tip-apex distance PO (mm)-min/max		9.85/35.9	6.18/37.5	
Tip-apex distance H (mm)-mean		17.49	19.45	
Tip-apex distance H (mm)-median		16.44	18.85	
Tip-apex distance H (mm)-min/max		10.6/35.6	4.6/38.8	
Tip-apex distance PO-H (mm)-median		0.29	0.34	*p* = 0.751
				
Blade prominance PO-H (mm)-mean	−5.52			*p* < 0.001 (Welch)
Blade prominance PO-H (mm)-median	−2.9			
				
Varotizazion- CCD PO- CCD H-mean	2.57°			*p* = 0.789 (Mann-Whitney U)
Varotizazion- CCD PO- CCD H-median	1.8°			
Varotizazion- CCD PO- CCD H-mean		2.67°	2.53°	
Varotizazion- CCD PO- CCD H-median		1.8°	1.9°	
				
Decreased varotization risk		NO		*p* = 0.245 (Fisher)
				
Varus deformity 5–10°	35	4	31	
Varus deformity > 10°	8	4	4	
Varotization risk optimal/suboptimal blade position	x			*p* = 0.690 (Fisher)
Varotization risk suboptimal blade position		x		*p* = 0.245 (Fisher)
Varotization risk optimal blade position		x		*p* = 0.179 (Fisher)
Varotization risk stable/unstable fracture type	x			*p* = 1.000 (Fisher)
Varotization risk unstable fracture type		x		*p* = 0.101 (Fisher)
Varotization risk stable fracture type		x		*p* = 0.396 (Fisher)
Cut-out	3	0	3	
